# Person-centered strategies for delivering TB diagnostic services in Lima, Peru

**DOI:** 10.5588/pha.23.0036

**Published:** 2023-09-21

**Authors:** C. M. Yuen, A. K. Millones, D. Acosta, I. Torres, S. Farroñay, J. Jimenez, L. Lecca

**Affiliations:** 1 Division of Global Health Equity, Brigham and Women’s Hospital, Boston, MA, USA;; 2 Department of Global Health and Social Medicine, Harvard Medical School, Boston, MA, USA;; 3 Socios En Salud Sucursal Peru, Lima, Peru

**Keywords:** patient preference, delivery of health care, community health services

## Abstract

**SETTING:**

Lima, Peru.

**OBJECTIVE:**

To close the gap in TB diagnosis, TB diagnostic services must match care-seeking preferences. We sought to identify preferred strategies for delivering TB diagnostic services and to determine whether preferences differ among demographic groups.

**DESIGN:**

During May 2022–January 2023, we recruited adults who recently initiated treatment for pulmonary TB. We used an object-case best-worst scaling instrument to assess the desirability of nine hypothetical strategies for delivering TB diagnostic services. A *t*-test was used to assess differences in preference scores between groups.

**RESULTS:**

Among 150 participants, the strategies with the highest preference scores were an integrated mobile unit offering screening for multiple conditions, expedited attention at the health center, and home-based screening. These were strongly preferred by 42%, 25%, and 27% of participants, respectively, and 80% of participants strongly preferred at least one of the three. Expedited attention at the health center scored more highly among people who experienced >2 months delay in TB diagnosis compared to those who experienced a more rapid diagnosis (0.37 ± 0.06 vs. 0.17 ± 0.06; *P* = 0.031).

**CONCLUSION:**

Providing person-centered TB diagnostic services at diverse access points could help reach different populations, which could promote early diagnosis and help close the diagnosis gap.

## Abstract (fr)

**CONTEXTE:**

Lima, Pérou.

**OBJECTIF:**

Pour combler les lacunes dans le diagnostic de la TB, les services de diagnostic de la TB doivent correspondre aux préférences en matière de recherche de soins. Nous avons cherché à identifier les stratégies préférées pour la prestation de services de diagnostic de la TB et à déterminer si les préférences diffèrent entre les groupes démographiques.

**MÉTHODE:**

De mai 2022 à janvier 2023, nous avons recruté des adultes ayant récemment commencé un traitement contre la TB pulmonaire. Nous avons utilisé un instrument d’évaluation du meilleur et du pire pour évaluer la désirabilité de neuf stratégies hypothétiques de prestation de services de diagnostic de la TB. Un test-*t* a été utilisé pour évaluer les différences dans les scores de préférence entre les groupes.

**RÉSULTATS:**

Parmi les 150 participants, les stratégies ayant les scores de préférence les plus élevés étaient une unité mobile intégrée offrant un dépistage pour des conditions multiples, une attention accélérée au centre de santé et un dépistage à domicile. Ces stratégies ont été fortement préférées par 42%, 25% et 27% des participants, respectivement, et 80% des participants ont fortement préféré au moins l’une des trois. L’attention accélérée au centre de santé a obtenu un score plus élevé chez les personnes ayant subi un retard de diagnostic de la TB de plus de 2 mois que chez celles ayant subi un diagnostic plus rapide (0,37 ± 0,06 contre 0,17 ± 0,06 ; *P* = 0,031).

**CONCLUSION:**

La fourniture de services de diagnostic de la TB centrés sur la personne à divers points d’accès pourrait permettre d’atteindre différentes populations, ce qui pourrait favoriser un diagnostic précoce et contribuer à combler le fossé en matière de diagnostic.

An estimated 30% of people with TB worldwide have historically gone untreated, with even larger gaps reported during the initial years of the COVID-19 pandemic.^1^ One contributor to underdiagnosis is that in many health systems, the locations of TB diagnostic services are not matched to the healthcare-seeking preferences of the population.^2^ For example, in Pakistan and Indonesia, the majority of people first seek care in the private sector, where TB diagnostic services are very limited.^3^,^4^ In Ethiopia, a third of people first seek care with health extension workers in the community, but no TB diagnostic services are available in community settings.^5^

To close the gap in diagnosis and provide person-centered care that is tailored to the needs of the individual, it is necessary to match TB diagnostic service delivery strategies to care-seeking preferences. Preferences and care-seeking behaviors likely vary among the population. For example, a study in rural China found that men experiencing prolonged cough were more likely than women to seek care first at a hospital, while women preferred to seek care first at drugstores or village clinics.^6^ A study in Zambia found that some people prioritized most the speed of TB diagnostic service delivery, while others prioritized confidentiality and privacy.^7^ Thus, person-centered TB diagnostic services ideally comprise a combination of delivery strategies, considering not only delivery location but how services are delivered.

Peru is a middle-income country with an estimated TB incidence of 130 per 100,000 population.^1^ TB services for diagnosis and treatment are available in public primary care health centers. For TB diagnosis, all primary care centers offer evaluation by a general medicine doctor and sputum collection for smear microscopy; some higher-level centers offer chest radiography. In this study, we sought to identify preferred strategies for delivering TB diagnostic services in Lima, Peru, and to determine whether the desirability of strategies differed among demographic groups.

## STUDY POPULATION, DESIGN, AND METHODS

### Study population

During May 2022–January 2023, we recruited adults (≥18 years old) who recently initiated treatment for pulmonary TB in the 12 primary care health centers that serve the district of Carabayllo in Lima, Peru. We limited the study to adult pulmonary TB as this type of TB is most amenable to being diagnosed outside health facilities, and different strategies are likely necessary for delivering diagnostic services to children and people with extrapulmonary TB.

### Study design

We conducted a cross-sectional survey with an object-case best–worst scaling (BWS) instrument to enable participants to rate a set of strategies for delivering TB diagnostic services. BWS is a stated preference survey method based on random utility theory, which assumes that the number of times that one item is chosen over another indicates the difference in participants’ preferences for the two items.^8^ BWS offers better discriminatory power and robustness compared to asking participants to rate the desirability of individual items using rating scales.^8^ We used object-case BWS as opposed to related discrete choice experiment methods because the options explored represented a range of conceptually different strategies rather than different combinations of a set of attributes.

### Survey development and administration

CMY and AKM defined the strategies presented in the survey based on their knowledge of existing TB diagnostic services, as well as interviews previously completed for another study in which people with TB were asked for suggestions to improve TB detection in their communities.^9^ While we selected strategies that we thought would be feasible to implement in the Peruvian health system, we did not consider the costs or logistics of implementation, as we wished the preferences of people with TB to drive our recommendations for service delivery. Nine delivery strategies were defined: three were based in health centers, five in the community, and one in institutions such as workplaces or schools (Table 1). We prepared a standardized description for each strategy to ensure consistency in descriptions presented to participants and to reduce the possibility that study staff might unconsciously suggest their own preferences to participants while describing the strategies. We also prepared a cartoon illustration to enable comprehension without requiring literacy. We used a balanced incomplete block design to create a survey with 12 choice sets of three options each, with each strategy appearing four times. Two versions of the survey were created with choice sets in the opposite order to ensure that fatigue did not systematically affect certain questions.

**TABLE 1:** Strategies for delivering TB diagnostic services presented in best-worst scaling surveys

Strategy	Description presented to participant	Service delivery location
Pulmonologist at health center	Ability to see a pulmonologist who comes to your health center one day a week	Health center
Expedited attention at health center	Rapid attention at the health center for people with TB symptoms; people with symptoms have a special direct entry point for medical attention	Health center
Private clinic voucher	Voucher to receive a free X-ray and medical evaluation at a private clinic	Health center
Screening unit near home	Mobile TB screening unit with X-ray close to your home, Tuesday–Sunday 8 am to 5 pm	Community
Screening unit in central location	Mobile TB screening unit with X-ray in a central location (e.g., the market), Tuesday–Sunday 8 am to 5 pm	Community
Integrated screening unit	Mobile unit that offers screening for TB, COVID-19, diabetes, hypertension, and mental health in a central location, Tuesday–Sunday 8 am to 5 pm	Community
Institutional campaign	TB screening campaign in collaboration with your place of work, school, or church, in which everyone at the institution is screened on a particular day	Institutional
Community campaign	TB screening campaign in collaboration with your neighborhood leadership association, offering TB education and screening during a weekend	Community
Home-based screening	A field team brings a portable X-ray machine to your home to perform a TB screening during a time that is convenient to you	Community

At the beginning of the survey, study staff presented each strategy using the illustration, then asked the participant to describe the strategy in their own words to ensure comprehension. Participants were instructed to think about the time prior to their TB diagnosis and consider which strategies they would have wanted the most during that period to help them be diagnosed more rapidly or conveniently. For each choice set, study staff placed the illustrations corresponding to three options on a table in front of the participant and asked the participant to select the ones that they preferred most and least. Study staff recorded participants’ answers. We also collected data on participant demographic and socioeconomic characteristics. Following survey administration, study staff asked participants an open-ended question about whether they had any comments about the strategies presented and noted their responses in a field note log.

### Analysis

We used a count-based method to calculate preferences scores for each strategy by subtracting the number of times it was chosen as the least preferred option from the number of times it was chosen as the most preferred option and dividing by the number of times it appeared in the survey.^10^ Possible scores range from –1.00 (least preferred) to +1.00 (most preferred). We averaged the scores of the five community-based strategies and the scores of the three health facility-based strategies to create combined preference scores for these sets of strategies. We considered a score of ⩾0.75 to indicate a strongly preferred strategy and a score of ⩽–0.75 to indicate a strongly disfavored strategy. We consulted the field note log to determine motivations behind preferences.*t*-tests were used to determine whether preference scores for each strategy differed by participant characteristics. We assessed differences by sex, age group (18–25 vs. >25 years, and 18–40 vs. >40 years), occupation (working or studying vs. no occupation, with a sensitivity analysis excluding students), whether the participant reported personal income, whether there were children in the household, the delay in diagnosis experienced (>2 months vs. ⩽2 months, excluding those who could not recall symptoms or time of symptom onset), and the level of health facility^11^ that the participant attended (level 4 vs. levels 2/3). *P* < 0.05 was considered statistically significant.

We performed latent class analysis to identify groups of participants with different patterns of preference scores for the nine individual strategies. We assessed models considering all nine strategies, as well as models that considered only five strategies to reduce correlation among preference scores. The Akaike Information Criterion and Bayesian Information Criterion were used to assess model fit. Analyses were performed in SAS v9.4 (SAS Institute, Cary, NC, USA).

### Ethics statement

Written informed consent was obtained from all participants. This study was approved by the institutional review boards at Mass General Brigham, Boston, MA; and the Universidad Peruana Cayetano Heredia, Lima, Peru.

## RESULTS

We enrolled 150 people who recently initiated treatment for pulmonary TB. Of these, 86 (57%) were male, 96 (64%) were 18–40 years old, and 87 (58%) were not working (Table 2). Participants’ median household size was 5 individuals (interquartile range 3–7), and 3 (2%) participants lived in congregate settings. TB diagnosis within 2 months of symptom onset was reported by 63 (42%) of participants.

**TABLE 2:** Characteristics of 150 participants who recently initiated treatment for pulmonary TB

Characteristics		*n* (%)
Sex	Female	64 (43)
	Male	86 (57)
Age group, years	18–25	46 (31)
	26–40	50 (33)
	41–55	32 (21)
	>46	22 (15)
Employment status	Works in formal sector	11 (7)
	Works in informal sector	41 (27)
	Student	11 (7)
	No occupation	87 (58)
Personal income	At least monthly minimum wage	37 (25)
	Less than monthly minimum wage	13 (9)
	Not working or declined answering	100 (67)
Adults in household (including participant)	1–2	35 (23)
	3–4	65 (43)
	⩾5	47 (31)
	Resident of congregate setting	3 (2)
Children in household	0	42 (28)
	1–2	76 (51)
	⩾3	32 (21)
Recalled symptoms prior to TB diagnosis	Yes	129 (86)
	No	21 (14)
Time between symptom onset and diagnosis, months	0–2	63 (42)
	>2	53 (35)
	Did not recall symptoms or onset date	34 (23)
Health care facility level*	Level 2	31 (21)
	Level 3	75 (50)
	Level 4	44 (29)

*Level 2 facilities offer outpatient general medicine services. Level 3 facilities offer outpatient general medicine, laboratory, and dentistry services. Level 4 facilities offer general medicine, laboratory, radiology, pediatrics, dentistry, psychology, and nutrition services, as well as delivery of uncomplicated pregnancies with capacity for overnight stays; they have longer hours than Level 2/3 facilities and receive referrals from Level 2/3 facilities for specialty services.

The strategies for delivering TB screening services with the highest preference scores were an integrated mobile unit offering screening for multiple conditions, expedited attention at one’s health center, and home-based screening (Figure). These strategies were strongly preferred by 42%, 25%, and 27% of participants, respectively, and 120 (80%) participants strongly preferred at least one of these strategies. The strategies with the lowest preference scores were one-day screening campaigns in collaboration with either the neighborhood leadership association or one’s workplace, school, or church. Almost every strategy had participants who strongly preferred it, as well as those who strongly disfavored it.

**FIGURE:**
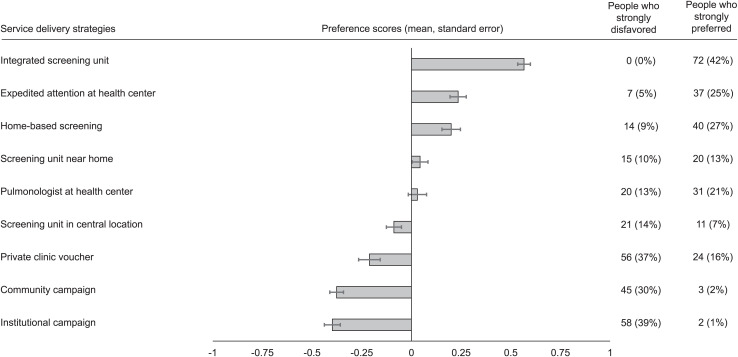
Preference scores for nine approaches to delivery of TB diagnostic services among 150 people who recently initiated TB treatment. ‘Strongly preferred’ was defined as preference scores of ⩾0.75, and ‘Strongly disfavored’ as preference scores of ⩽–0.75.

The field note log contained comments from 43 (29%) participants. The most common comment was that pulmonologists need to come to the primary care health centers more than the one day a week presented in the survey (10 participants). In addition, 10 participants mentioned discrimination as a motivation for disfavoring certain strategies; they felt that clients with vouchers would be discriminated against at private clinics compared to clients paying out of pocket (*n* = 5), that neighbors would discriminate against them if they were seen attending mobile screening units near their home or welcoming a screening team into their home (*n* = 4), and that a dedicated line for expedited attention in the health facility could subject people with respiratory symptoms to discrimination (*n* = 1).

People over 25 years old had significantly higher preference scores for community-based interventions than people aged 18–25 years (mean 0.09 ± standard error [SE] 0.02 vs. 0.01 ± SE 0.03; *P* = 0.042). Conversely, people aged 18–25 years had higher preference scores for health facility-based interventions, but this difference was not statistically significant (0.09 ± SE 0.05 vs. –0.01 ± SE 0.03; *P* = 0.098). People over 40 years old had higher preference scores for a pulmonologist in their health center than people under 40 (0.15 ± SE 0.08 vs. –0.04 ± SE 0.06; *P* = 0.045). Expedited attention at the health center scored more highly among people who attended a level 4 health center than those at lower-level health centers (0.38 ± SE 0.06 vs. 0.17 ± SE 0.05; *P* = 0.021), as well as among people who experienced >2 months delay in TB diagnosis than those who had a more rapid diagnosis (0.37 ± SE 0.06 vs. 0.17 ± SE 0.06; *P* = 0.031). No other characteristics assessed were associated with differences in preference scores for any strategy. In the latent class analysis, a one-class model exhibited the best fit.

## DISCUSSION

Our results suggest that programs need to utilize a variety of strategies for delivering TB diagnostic services in order to reach all people with TB promptly and efficiently. We observed heterogeneity and individualized preferences among study participants, and no single strategy was strongly preferred by everyone. Indeed, while all of the options in our survey were strongly preferred by at least one person, most were strongly disfavored by a substantial proportion of participants. The three most desirable strategies represented three different points of care delivery (community, facility, home); while each individual strategy was strongly preferred by less than half of participants, 80% of participants strongly preferred at least one of the three. The absence of latent classes suggests that people’s preferences are individual and may not fall into predictable patterns. Thus, early diagnosis could be supported by enabling access to TB diagnostic services at diverse points of care, which are likely to reach different populations.

The most preferred strategy in our study was integrated community-based services that screen for multiple conditions. Advantages of integrated screening include convenience and efficiency for clients, as well as reduced stigma if screening for relatively non-stigmatized conditions like hypertension is combined with screening for stigmatized conditions such as TB or HIV.^12^ Given the concerns about discrimination voiced by multiple participants, it is possible that stigma reduction promoted preference for this strategy in our study. While it is already feasible in many places to offer community-based screening for TB and other conditions at the same time, it can be challenging to ensure linkage to care in health systems where different clinics or providers manage TB, other infectious diseases, and non-communicable diseases.^13^ Ideally, primary healthcare systems will be strengthened to provide person-centered, integrated care for common non-communicable and infectious diseases, including TB. This will require de-fragmentation of donor funding, which currently supports single-disease programs,^14^ and would be facilitated by electronic medical record systems that allow different providers to see a patient’s entire medical record.

Expedited attention at health facilities, the second most preferred intervention overall, was particularly valued by people who had experienced longer delays in diagnosis. Long wait times can be a deterrent to care-seeking, and people with TB have reported delaying care-seeking due to work or caregiver duties.^15^,^16^ Similar to our findings, a discrete choice experiment among people with TB in Zambia found that those who prioritized speed of service (e.g., shorter wait times, same-day results) were more likely to have experienced longer delays.^7^ Programs to expedite TB evaluations among people who are hospitalized have been shown to improve TB detection and reduce delays to diagnosis.^17^,^18^ In general outpatient settings, offering expedited attention to people with respiratory symptoms without compromising the timeliness of care for people without symptoms would require adding additional providers who are trained in diagnosing respiratory conditions.

The major limitation of our study is that we surveyed people who had been diagnosed with TB about what services they hypothetically would have preferred before their diagnosis. Including only people with who initiated treatment fails to capture the preferences of people with TB who are not diagnosed, who are, arguably, the most important group to reach. Furthermore, although we asked people to think about the time before they knew they had TB, preferences expressed post hoc might not accurately reflect the preferences they would have exhibited before diagnosis. However, given that TB is a relatively rare condition that is not randomly distributed within communities, people who were recently diagnosed with TB are the best proxy group for assessing the preferences of people who have TB but have not yet been diagnosed. Another limitation was the modest sample size, which is consistent with other BWS studies,^19^ but may have compromised our ability to detect latent classes. Finally, we did not conduct qualitative research around why participants liked or did not like certain strategies, although we gained some insight into participants’ motivations through open-ended questioning at the end of the survey.

In conclusion, person-centered TB diagnostic services must accommodate heterogeneous preferences in how people with TB want to access care. Our findings suggest that in Lima, Peru, a three-pronged approach of offering integrated screening services for TB and other conditions in communities, expediting diagnostic services in health facilities, and offering home-based screening would provide the majority of people with TB with a desirable option for accessing diagnostic services. This type of structured effort to assess preferences can inform program planning to ensure that TB diagnostic service delivery responds to the needs of people with TB. Making TB diagnostic services accessible in multiple types of locations and through multiple delivery strategies to accommodate different preferences will help promote early diagnosis and close the diagnostic gap.
